# JAK Inhibition in *PNPT1* ‐Related Mitochondrial Interferonopathy: A Case Report and Review of Mitochondrial–Immune Crosstalk

**DOI:** 10.1002/jmd2.70096

**Published:** 2026-06-29

**Authors:** Dan Ross Brooks, Hyun Yong Koh, Taylor Martin Kerrins, Steven Lang, Emily Bland, Rui Yang, Sarah Kogan Nicholas, Stephanie Jean Sikkink, Stephen Kralik, Christine Eng, Chaya Nautiyal Murali, Seema Lalani, Pilar Lenglet Magoulas, Lisa Emrick, Kristen Sydney Fisher, Fernando Scaglia

**Affiliations:** ^1^ Department of Molecular and Human Genetics Baylor College of Medicine Houston Texas USA; ^2^ Texas Children's Hospital Houston Texas USA; ^3^ Division of Neurology, Department of Pediatrics Baylor College of Medicine Houston Texas USA; ^4^ Division of Neurology Texas Children's Hospital Houston Texas USA; ^5^ Division of Allergy & Immunology, Department of Pediatrics Baylor College of Medicine Houston Texas USA; ^6^ Division of Allergy & Immunology Texas Children's Hospital Houston Texas USA; ^7^ Department of Pediatrics & Internal Medicine Geisinger Medical Center Danville Pennsylvania USA; ^8^ Department of Radiology Texas Children's Hospital Houston Texas USA; ^9^ Department of Radiology Baylor College of Medicine Houston Texas USA; ^10^ Baylor Genetics Houston Texas USA; ^11^ Joint BCM–CUHK Center of Medical Genetics Prince of Wales Hospital Hong Kong SAR China

**Keywords:** COXPD13, JAK inhibitor, mitochondrial interferonopathy, *PNPT1*, polynucleotide phosphorylase (PNPase), tofacitinib, type I interferon

## Abstract

Biallelic pathogenic variants in *PNPT1* cause combined oxidative phosphorylation deficiency 13 (COXPD13) (MIM #614932), linking mitochondrial dysfunction to type I interferon (IFN) activation through cytosolic leakage of mitochondrial double‐stranded RNA (mt‐dsRNA). This mechanism connects mitochondrial disease to interferonopathies such as Aicardi–Goutières syndrome (AGS). We describe a 7‐month‐old female infant with compound heterozygous *PNPT1* variants presenting with severe hypotonia, feeding difficulties necessitating gastrostomy, dystonia, and elevated serum lactate. Brain magnetic resonance imaging (MRI) demonstrated marked cerebellar, brainstem, and basal ganglia atrophy, with a lactate peak on MR spectroscopy (consistent with an inverted doublet). Serum immune profiling revealed a mild but elevated type I IFN signature. Given the mechanistic overlap with AGS, off‐label tofacitinib, a Janus kinase (JAK) inhibitor that blocks IFN‐driven JAK/STAT signaling, was initiated following pediatric interferonopathy dosing protocols. Tofacitinib was associated with normalization of serum type I IFN biomarkers, reduction in lactate and transaminases, improvement in dystonic movements, ventilatory stability, and improved growth/nutrition without treatment‐limiting adverse events. To our knowledge, this represents the first reported use of JAK inhibition in COXPD13. The observed clinical and biochemical stabilization supports defining COXPD13 as a “mitochondrial interferonopathy” and suggests that IFN‐signature screening may identify mitochondrial disease patients who could benefit from targeted immunomodulation.

## Key Points

- COXPD13 results from biallelic *PNPT1* variants that impair mitochondrial RNA processing, leading to cytosolic accumulation of mitochondrial double‐stranded RNA with subsequent melanoma differentiation‐associated protein 5 (MDA5)‐mediated type I IFN activation, a mechanism shared with canonical interferonopathies such as Aicardi‐Goutières syndrome (AGS).
- A peripheral type I IFN signature, here assessed using a flow‐cytometric CD14^+^CD169^+^ monocyte assay, can identify the inflammatory component of mitochondrial disease and provide a measurable biomarker to guide and monitor targeted therapy.
- In this infant, treatment with tofacitinib (a JAK inhibitor) normalized the IFN signature within 4 weeks and was associated with reductions in serum lactate and transaminases, improvement in dystonia and ventilatory stability, and catch‐up growth, without treatment‐limiting adverse events.
- IFN‐signature screening in mitochondrial disease may identify a subset of patients amenable to targeted immunomodulation; however, case‐level observations require validation in systematic studies before broader clinical adoption.

## 1 Introduction

Beyond their canonical roles in bioenergetics and apoptosis, mitochondria are now recognized as central regulators of innate immunity. Aberrant release of mitochondrial nucleic acids into the cytosol activates nucleic acid–sensing pathways that drive type I interferon (IFN) responses, a process that is tightly restrained under physiologic conditions [1, 2, 3, 4]. Polynucleotide phosphorylase (PNPase), encoded by *PNPT1*, mediates mitochondrial RNA import and turnover; biallelic *PNPT1* variants impair these processes, promoting accumulation and cytosolic leakage of mt‐dsRNA and MDA5‐dependent chronic type I IFN activation [1, 3, 5, 6, 7, 8, 9].

Clinically, COXPD13 spans neurodevelopmental and metabolic phenotypes with neuroimaging ranging from Leigh‐like basal ganglia lesions to cystic leukoencephalopathy, overlapping classic interferonopathies such as AGS [8, 10, 11, 12]. This intersection has crystallized the concept of “mitochondrial interferonopathies,” in which dysregulated IFN signaling and autoinflammation are central, and interferon‐stimulated gene (ISG) signatures correlate with disease activity [2, 3, 4, 13, 14, 15].

Therapeutically, JAK inhibitors attenuate JAK/STAT signaling downstream of type I IFN receptors and have shown benefit across monogenic interferonopathies, including improvements in cutaneous, neurologic, and systemic inflammation with biomarker normalization [13, 16, 17, 18, 19, 20]. However, optimal dosing, safety, treatment duration, and durability of clinical and biomarker response in mitochondrial disease populations remain incompletely defined.

In summary, COXPD13 illustrates dysfunctional mitochondria‐immune crosstalk where defective PNPase function triggers pathogenic IFN signaling. These mechanistic insights support the exploration of targeted immunomodulation with JAK inhibition in carefully selected individuals, while highlighting the importance of systematic studies of efficacy, safety, and population selection.

## 2 Case Report/Methods

### 2.1 Clinical Summary

The patient was a female infant, the first child of healthy, non‐consanguineous parents, born at 37 weeks' gestation after a pregnancy complicated by symmetric intrauterine growth restriction (birth weight 2205 g, < 1st percentile). She required a prolonged 56‐day neonatal intensive care unit (NICU) course for neonatal respiratory failure requiring intubation and continuous positive airway pressure (CPAP), hypotonia, and dysphagia with aspiration confirmed on swallow study. Gastroesophageal reflux necessitated Nissen fundoplication and gastrostomy tube (G‐tube) placement.

From birth, she exhibited global developmental delay, microcephaly, and abnormal movements concerning for a central neurologic process. She subsequently developed progressive hypotonia, poor visual tracking, and loss of motor milestones, including head control. By approximately 3 months of age, she presented after an apneic episode with family‐initiated cardiopulmonary resuscitation (CPR), arriving with poor respiratory effort, recurrent desaturations, and bradycardic spells. Despite noninvasive ventilatory support (CPAP/bilevel positive airway pressure [BiPAP]), she continued to have frequent desaturations and aspiration events. Tracheostomy was placed at approximately 4 months of age, and she became fully ventilator‐dependent. She exhibited intermittent dystonic posturing, opisthotonus with autonomic changes (neurostorming), and myoclonic jerks; continuous EEG confirmed these episodes were non‐epileptic. During evaluation for neurologic decline, she was markedly hypotonic, with a weak cry and suck, minimal antigravity movement, and growth parameters below the 3rd percentile. Recurrent episodes of apnea and desaturation required mechanical ventilation, and she eventually became ventilator‐dependent through a tracheostomy.

### 2.2 Neuroimaging

**FIGURE 1:**
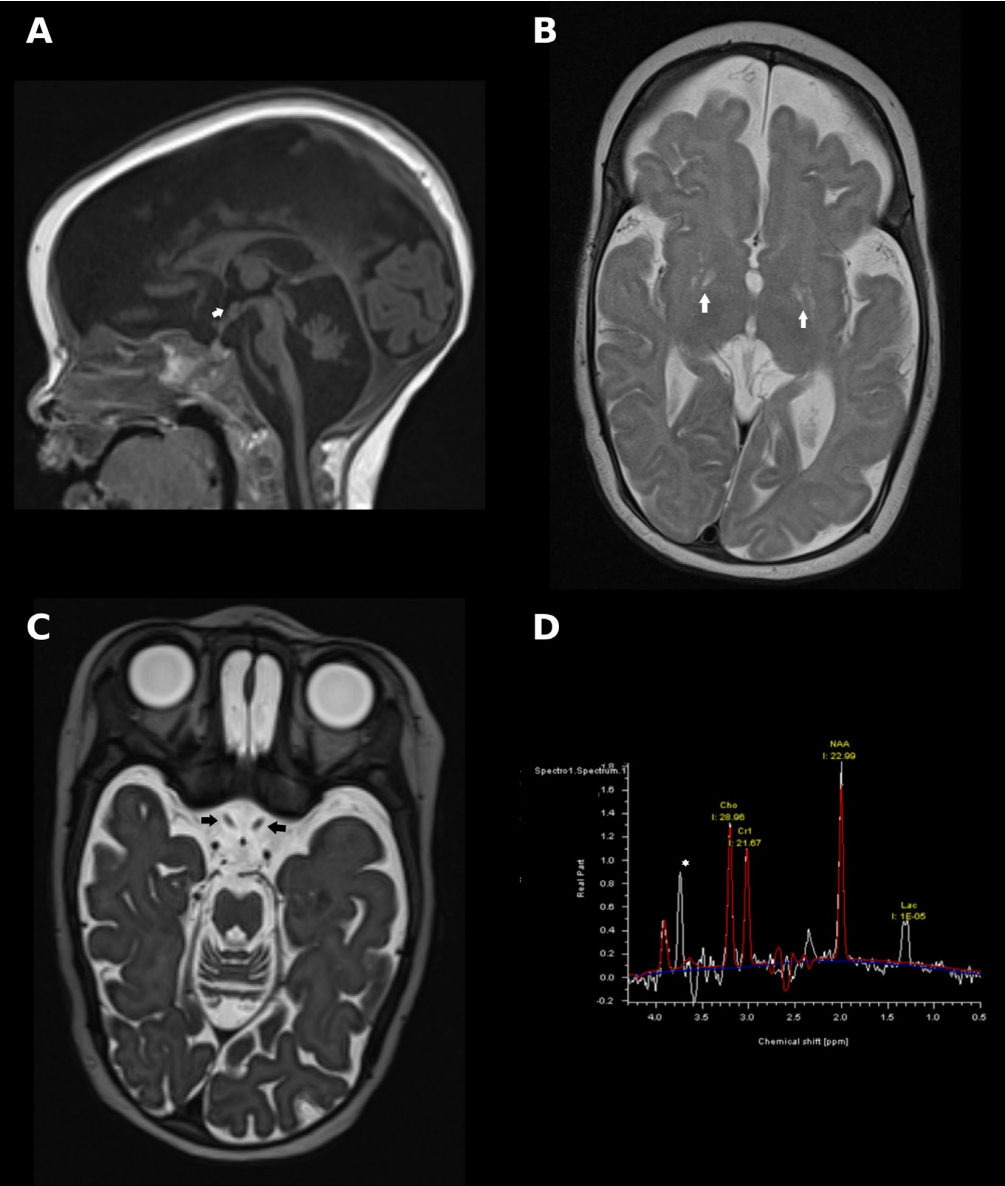
Brain MRI and Brain MR spectroscopy findings at 3 months of age. (A) Sagittal T1‐weighted image demonstrating severe cerebellar and brainstem volume loss, thinning of the corpus callosum, and a suspected hypothalamic adhesion (arrow). (B) Axial T2‐weighted image showing moderate cerebral and thalamic atrophy, and T2 hyperintensity in the globus pallidi (arrows). (C) Coronal T2‐weighted image demonstrating intracranial optic nerve volume loss (arrows). (D) Single‐voxel proton MR spectroscopy (TE = 270 ms) of the left basal ganglia showing a lactate doublet at ~1.3 ppm with preserved NAA, choline, and creatine peaks and an increased glutamate/glutamine peak at 3.75 ppm (asterisk).

Brain MRI at 3 months of age demonstrated severe volume loss of the cerebellum, brainstem, and intracranial optic nerves/chiasm, with moderate atrophy of the basal ganglia, thalami, and cerebral hemispheres, thinning of the corpus callosum, and a suspected hypothalamic adhesion on sagittal imaging (Figure 1A–C). On T2/fluid‐attenuated inversion recovery (FLAIR) sequences, there was no cystic leukoencephalopathy but mild symmetric signal abnormalities in globi pallidi typical of Leigh syndrome as reported in other COXPD13 cases (Figure 1B). Diffusion‐weighted imaging showed symmetric globus pallidus hyperintensity with corresponding apparent diffusion coefficient (ADC) hyperintensity, without evidence of acute restricted diffusion. Small bilateral subdural collections (2–5 mm) were attributed to ex vacuo changes. Single‐ and multi‐voxel proton MR spectroscopy demonstrated elevated lactate (doublet at ~1.3 ppm) across multiple regions, with preserved N‐acetylaspartate (NAA), choline, and creatine peaks and an additional peak at ~3.75 ppm consistent with glutamate/glutamine (Figure 1D). These findings are consistent with previously reported COXPD13‐related neuroimaging, although the COXPD13 phenotype is broad and has variably included Leigh‐pattern lesions, cystic leukoencephalopathy, temporal cysts, or normal imaging [7, 12]. Representative images are shown in Figure 1.

### 2.3 Genetic Testing

Trio whole‐genome sequencing (WGS) identified compound heterozygous variants in *PNPT1* (NM_033109.5). The first, c.680‐3T>A, is an intronic variant inherited maternally; SpliceAI predicted a possible splicing effect (score 0.160), and it was classified as likely pathogenic. The second, c.1297G>A (p.Ala433Thr), is a paternally inherited missense variant resulting in the substitution of alanine with threonine at codon 433. This residue is conserved across species, and in silico prediction tools (CADD = 26.9; REVEL = 0.723) suggest a potential deleterious effect on protein function; however, the variant remains classified as a variant of uncertain significance (VUS). Together, the two variants were confirmed in trans and are consistent with clinical suspicion of COXPD13. Variant interpretation was conducted in accordance with ACMG criteria [21] and supported by functional annotation and prior literature [7, 9].

### 2.4 Immunologic Testing

Given the overlap with interferonopathies, whole blood was analyzed for Type I and II IFN signatures by flow cytometry (T1A2MP assay, Nationwide Children's Hospital, Columbus, Ohio). This assay quantifies CD14^+^CD169^+^ monocytes using anti‐CD14 (clone M5E2) and anti‐CD169 (clone 7–239) antibodies, and PD‐L1 (CD274) expression as protein‐based surrogate markers of IFN activity. These are validated clinical biomarkers of type I interferonopathy, distinct from transcript‐based ISG expression assays [22, 23, 24]. Reference ranges: CD14^+^CD169^+^ monocytes < 3% (elevated ≥ 3%); mean fluorescence intensity (MFI) 1205–8343. The pre‐treatment sample demonstrated elevated CD14^+^CD169^+^ monocytes (76%; reference < 3%) with normal MFI (7241; reference 1205–8343), consistent with a mildly elevated type I IFN signature on this single pre‐treatment timepoint. CD274 (PD‐L1) expression was not elevated, indicating no type II IFN response. A post‐treatment sample collected at 4 weeks showed normalization of IFN parameters (CD14^+^CD169^+^ monocytes 2%, MFI 2933, within reference), consistent with resolution of the type I IFN signature following JAK inhibitor therapy.

### 2.5 Treatment Protocol

After multidisciplinary discussion involving Genetics, Neurology, Immunology, and Pulmonology, off‐label therapy with tofacitinib, a JAK inhibitor, was initiated at 3 months of age following confirmation of an elevated type I IFN signature. Treatment was initiated based on a mechanism‐driven, biomarker‐guided rationale: the patient demonstrated an elevated type I IFN signature, PNPT1 dysfunction is mechanistically linked to mt‐dsRNA–mediated IFN activation, and JAK inhibition has demonstrated efficacy in monogenic interferonopathies [18, 20, 25]. Importantly, the expected benefit was attenuation of IFN‐driven inflammation rather than correction of primary oxidative phosphorylation dysfunction. The team recognized that the neurologic response to JAK inhibition in conditions such as AGS may be limited [26] and counseled the family accordingly, with goals focused on biochemical and systemic stabilization.

Dosing was extrapolated from available pediatric experience in interferonopathies and juvenile idiopathic arthritis (JIA), with a conservative adjustment for age, weight, and organ function [18, 27, 28, 29, 30]. Specifically, based on established pediatric tofacitinib dosing for polyarticular course JIA (3.2 mg twice daily for children 10–20 kg, equivalent to approximately 0.64 mg/kg/day) [28, 30], the dose was conservatively extrapolated for this 4.2 kg infant. Therapy was initiated at approximately 0.4 mg/kg/day, divided twice daily, corresponding to a total daily dose of approximately 1.6 mg, and was maintained throughout the hospitalization. Formal pharmacokinetic evaluation was not performed. Immunization status was reviewed prior to initiation. The patient had not received her 2‐month immunizations at the scheduled visit due to a change in primary care providers. Age‐appropriate inactivated/subunit vaccines (DTaP, IPV, PCV13, Hib, and hepatitis B) were administered before starting tofacitinib. The oral rotavirus vaccine, a live attenuated vaccine, was also administered prior to tofacitinib initiation, as it was age‐indicated at 2 months and the patient had not yet begun immunosuppressive therapy. Live attenuated injectable vaccines (MMR and VZV), the concurrent use of which should be avoided during tofacitinib therapy per the Summary of Product Characteristics, were not yet age‐indicated at treatment initiation (3 months), as these are not scheduled until 12 months of age per the Advisory Committee on Immunization Practices/Centers for Disease Control and Prevention (ACIP/CDC) immunization schedule.

Concomitant supportive care included invasive mechanical ventilation via tracheostomy and gastrostomy‐based nutritional support; no additional immunomodulatory therapies were administered concurrently. Baseline and serial monitoring included a complete blood count with differential, comprehensive metabolic panel with hepatic and renal function assessment, lipid profile, and infection surveillance, in accordance with published safety recommendations for pediatric JAK inhibitor use [27, 28]. The patient remained clinically stable during the inpatient treatment period, with no treatment‐limiting adverse effects identified.

### 2.6 Clinical and Biochemical Monitoring

Clinical response was assessed through serial neurological examinations, documentation of feeding and ventilatory requirements, and growth parameters. Biochemical monitoring included serial serum lactate, serum transaminases, and serum IFN activity measured by the flow cytometry–based assay. Adverse events were tracked systematically, with particular attention to infection risk, cytopenias, and hepatic or renal dysfunction [27, 28, 31].

## 3 Results

Following initiation of tofacitinib at an infant‐adjusted dose of 0.4 mg/kg/day, the patient demonstrated marked clinical and biochemical improvement (Table 1). Type I IFN activity, as measured by a flow cytometry–based assay (CD14^+^CD169^+^ monocyte expression), normalized within 4 weeks of therapy, consistent with the expected pharmacodynamic effect of JAK inhibition in interferonopathies [18, 20, 32, 33]. Progressive clinical improvement was observed over subsequent weeks following this initial biochemical response.

**TABLE 1:** Clinical and immunologic response to tofacitinib therapy.

	Pre‐treatment	Post‐treatment	Reference range
Interferon signature (monocytes)	Mild Type I IFN+ (CD14^+^CD169^+^:76%)^a^	Normal (CD14^+^CD169^+^: 2%)	CD14^+^CD169^+^ < 3%
AST	88 U/L	57 U/L	20–60 U/L
ALT	73 U/L	46 U/L	14–45 U/L
Lactate	7.9 mmol/L	2.3 mmol/L	0.2–1.7 mmol/L
Blood gas	pH 7.25/pCO_2_ 63	pH 7.38/pCO_2_ 46	7.32–7.42/40–50 mmHg
Respiratory support	Full ventilatory support with tracheostomy	Tolerating home ventilation (CPAP/BiPAP)	—
Neurologic status	Myoclonic jerks, dystonia	Improved abnormal movements and tone	—
Nutritional status (WAZ)	Z‐score −3.36	Z‐score −2.18	—
CBC	Hgb 10.6 g/dL; Plt 587 × 10^9^/L; WBC 10.5 × 10^9^/L; ANC 5.2 × 10^9^/L; no cytopenias	Hgb 11.7 g/dL; Plt 833 × 10^9^/L; WBC 11.1 × 10^9^/L; ANC 5.2 × 10^9^/L; no cytopenias	Hgb ≥ 11.5 g/dL; ANC ≥ 1.5 × 10^9^/L; Plt 150–400 × 10^9^/L

Abbreviations: ALT, alanine aminotransferase; ANC, absolute neutrophil count; AST, aspartate aminotransferase; BiPAP, bilevel positive airway pressure; CBC, complete blood count; CPAP, continuous positive airway pressure; Hgb, hemoglobin; IFN, interferon; Plt, platelets; WAZ, weight‐for‐age z‐score; WBC, white blood cell count.

^a^ CD14^+^CD169^+^ monocytes represent a flow cytometry–based marker of type I interferon activity.

Serum lactate improved substantially from a pre‐treatment level of 7.9 mmol/L to a nadir of 2.3 mmol/L, with concomitant decreases in liver transaminases (AST nadir 57 U/L; ALT nadir 46 U/L), reflecting improved mitochondrial and hepatic function. Liver transaminases remained mildly to moderately elevated on serial monitoring (AST 64–117 U/L, ALT 64–106 U/L, GGT 97–192 U/L) without hyperbilirubinemia or evidence of hepatic synthetic dysfunction. This pattern was deemed consistent with underlying mitochondrial disease and/or medication exposure (tofacitinib) and did not warrant treatment discontinuation. Pre‐existing transaminase elevations also remained stable during a subsequent course of remdesivir for COVID‐19 pneumonia, with no progression of hepatic injury. Serum bilirubin and albumin remained normal throughout. Serial complete blood counts demonstrated no treatment‐emergent cytopenias at any time point. Specifically, hemoglobin remained stable (10.6–11.7 g/dL), with no neutropenia (ANC nadir 5.2 × 10^9^/L) or lymphopenia. Thrombocytosis was noted (platelets 587–833 × 10^9^/L), attributed to reactive thrombocytosis in the setting of chronic inflammation and intercurrent illness rather than a treatment‐related adverse effect. Monocytosis was also observed (16.5%; absolute monocyte count 1.82 × 10^9^/L), consistent with chronic inflammatory activation. C‐reactive protein was not significantly elevated (< 0.5 mg/dL).

The patient had no serious opportunistic infections attributable to tofacitinib. She developed bacterial tracheitis with *Staphylococcus aureus* and subsequently *Pseudomonas aeruginosa* on tracheal aspirates during the post‐tracheostomy period, treated with targeted intravenous antibiotics and inhaled tobramycin. At approximately 6 months of age, she developed COVID‐19 pneumonia with acute‐on‐chronic hypoxemic and hypercarbic respiratory failure, managed with dexamethasone, remdesivir, and augmented airway clearance, with clinical recovery by approximately 7 months of age. These infectious events are consistent with her degree of medical complexity and ventilator dependence rather than JAK inhibitor‐attributable immunosuppression, as no opportunistic or unusual pathogens were identified.

Dystonic movements and myoclonic jerks were substantially reduced, with improved respiratory stability and fewer desaturation episodes. Weight and length percentiles improved, reflecting catch‐up growth. No treatment‐limiting adverse events, cytopenias, hepatic synthetic dysfunction, or opportunistic infections were observed. The patient remains on ongoing JAK inhibitor therapy with continued clinical stability. These outcomes, including normalization of IFN biomarkers, improved organ function and growth, and a favorable safety profile, align with prior pediatric interferonopathy cohorts treated with JAK inhibitors [18, 20, 32, 34, 35], though ongoing monitoring remains essential.

## 4 Discussion

COXPD13 is a *PNPT1*‐related mitochondrial disease that exemplifies the convergence of mitochondrial dysfunction and dysregulated innate immune signaling. Biallelic *PNPT1* variants impair degradation and compartmentalization of mt‐dsRNA, resulting in cytosolic escape and aberrant MDA5‐dependent antiviral signaling. This aberrant antiviral signaling drives chronic type I IFN activation, a mechanism increasingly recognized in both classic interferonopathies and a subset of mitochondrial disorders [1, 3, 12, 36].

Neuroimaging in COXPD13 is heterogeneous, ranging from Leigh‐like basal ganglia lesions to cystic leukoencephalopathy resembling AGS and RNASET2 deficiency [5, 12]. This radiologic overlap underscores the shared axis of nucleic acid–driven IFN activation and highlights the clinical value of screening for IFN signatures in children with unexplained encephalopathy and atypical imaging ([3, 5, 12]). The concept of “mitochondrial interferonopathies” has emerged to capture this intersection, with increasing evidence that mitochondrial nucleic acid release can drive autoinflammatory disease states previously attributed solely to canonical interferonopathies [1, 3, 13]. In addition to mt‐dsRNA leakage seen in COXPD13, pathogenic *ATAD3A* variants have been shown to cause release of mitochondrial DNA into the cytoplasm, leading to cGAS–STING–mediated type I interferon activation [37]. This underscores that distinct mitochondrial nucleic acid species—RNA in COXPD13 and DNA in ATAD3A deficiency—can converge on a shared IFN‐driven inflammatory pathway.

Therapeutically, JAK inhibitors such as tofacitinib and baricitinib have demonstrated efficacy in reducing IFN‐driven inflammation in monogenic interferonopathies, including AGS, Sting‐associated vasculopathy with onset in infancy (SAVI), and spondyloenchondrodysplasia with immune dysregulation (SPENCD) [18, 20, 25, 38]. In our patient, tofacitinib was associated with rapid normalization of interferon biomarkers and improvements in dystonic movements, ventilatory status, and growth trajectory, with no major adverse events observed. These results mirror published case series and in vitro studies supporting the safety and potential disease‐modifying effects of JAK inhibition in interferonopathies [18, 20, 39, 40]. To our knowledge, this represents the first reported use of JAK inhibition in COXPD13, extending the therapeutic paradigm of interferonopathies into the mitochondrial field.

Several limitations must be acknowledged. As a single case report, generalizability is restricted, and spontaneous stabilization cannot be excluded, as occasionally occurs in autoinflammatory encephalopathies and other mitochondrial diseases [41, 42, 43]. One of the identified *PNPT1* variants remains classified as a VUS, although segregation, evolutionary conservation, and clinical correlation collectively support its pathogenicity. Functional studies, such as PNPT1 western blotting in patient‐derived cells, were not performed, representing an additional limitation in establishing variant pathogenicity.

The interferon signature was only mildly elevated on a single pre‐treatment occasion, raising questions about thresholds for intervention and the spectrum of mitochondrial disease responsive to JAK inhibition. Follow‐up brain MRI was not obtained, as the neurology team determined that the risks of sedation outweighed the potential clinical benefit in this medically fragile, ventilator‐dependent infant; neuroimaging response to JAK inhibition, therefore, cannot be assessed. Broader cytokine profiling was not performed. Given the pleiotropic effects of tofacitinib on multiple cytokine signaling pathways beyond type I IFN, the observed clinical improvement cannot be attributed solely to IFN pathway modulation. Finally, follow‐up remains short, limiting assessment of long‐term efficacy, neurodevelopmental outcomes, and late adverse effects. Formal pharmacokinetic evaluation was not conducted. The patient experienced infectious complications (bacterial tracheitis and COVID‐19 pneumonia) during tofacitinib therapy; while attributable to her underlying medical fragility and ventilator dependence rather than opportunistic immunosuppression, these events highlight the importance of careful risk stratification and infection monitoring in medically complex infants receiving JAK inhibitor therapy.

In summary, this case highlights the mechanistic and clinical overlap between mitochondrial disease and interferonopathies and supports the emerging paradigm of mitochondrial interferonopathies. Our findings suggest that targeted immunomodulation with JAK inhibitors may offer therapeutic benefit in select patients, but systematic studies are needed to define patient selection, treatment protocols, and long‐term outcomes.

## Author Contributions

**Dan Ross Brooks:** conceptualization, data curation, investigation, writing – original draft. **Hyun Yong Koh, Taylor Martin Kerrins, Steven Lang, Emily Bland, Rui Yang, Stephanie Jean Sikkink:** investigation, writing – review and editing. **Sarah Kogan Nicholas, Christine Eng, Pilar Lenglet Magoulas, Lisa Emrick, Kristen Sydney Fisher, Fernando Scaglia:** supervision, interpretation, writing – review and editing. **Stephen Kralik:** resources, investigation, interpretation.

## Funding

This work was supported in part by the American College of Medical Genetics (ACMG) Foundation Award, awarded to Dr. Dan Brooks.

## Ethics Statement

This study was conducted in accordance with the Declaration of Helsinki.

## Consent

Informed consent for participation and publication was obtained from the patient's legal guardians.

## Conflicts of Interest

The authors declare no conflicts of interest.

## Data Availability

The data that support the findings of this study are available from the corresponding author upon reasonable request.

## References

[1] A. Dhir , S. Dhir , L. S. Borowski , et al., “Mitochondrial Double‐Stranded RNA Triggers Antiviral Signalling in Humans,” Nature 560, no. 7717 (2018): 238–242, 10.1038/s41586-018-0363-0. 30046113 PMC6570621

[2] N. Keshavan , L. Mhaldien , K. Gilmour , and S. Rahman , “Interferon Stimulated Gene Expression Is a Biomarker for Primary Mitochondrial Disease,” Annals of Neurology 96, no. 6 (2024): 1185–1200, 10.1002/ana.27081. 39320038

[3] A. Lepelley , T. Wai , and Y. J. Crow , “Mitochondrial Nucleic Acid as a Driver of Pathogenic Type I Interferon Induction in Mendelian Disease,” Frontiers in Immunology 12 (2021): 729763, 10.3389/fimmu.2021.729763. 34512665 PMC8428523

[4] A. P. West and P. J. McGuire , “Tipping the Balance: Innate and Adaptive Immunity in Mitochondrial Disease,” Current Opinion in Immunology 95 (2025): 102566, 10.1016/j.coi.2025.102566. 40424975 PMC12210220

[5] D. Bamborschke , M. Kreutzer , A. Koy , et al., “PNPT1 Mutations May Cause Aicardi‐Goutières‐Syndrome,” Brain and Development 43, no. 2 (2021): 320–324, 10.1016/j.braindev.2020.10.005. 33158637

[6] C. G. Hsu , W. Li , M. Sowden , C. L. Chávez , and B. C. Berk , “Pnpt1 Mediates NLRP3 Inflammasome Activation by MAVS and Metabolic Reprogramming in Macrophages,” Cellular & Molecular Immunology 20, no. 2 (2023): 131–142, 10.1038/s41423-022-00962-2. 36596874 PMC9886977

[7] S. Matilainen , C. J. Carroll , U. Richter , et al., “Defective Mitochondrial RNA Processing due to PNPT1 Variants Causes Leigh Syndrome,” Human Molecular Genetics 26, no. 17 (2017): 3352–3361, 10.1093/hmg/ddx221. 28645153

[8] R. Sato , N. Arai‐Ichinoi , A. Kikuchi , et al., “Novel Biallelic Mutations in the *PNPT1* Gene Encoding a Mitochondrial‐RNA‐Import Protein PNPase Cause Delayed Myelination,” Clinical Genetics 93, no. 2 (2018): 242–247, 10.1111/cge.13068. 28594066

[9] V. Vedrenne , A. Gowher , P. De Lonlay , et al., “Mutation in PNPT1, Which Encodes a Polyribonucleotide Nucleotidyltransferase, Impairs RNA Import Into Mitochondria and Causes Respiratory‐Chain Deficiency,” American Journal of Human Genetics 91, no. 5 (2012): 912–918, 10.1016/j.ajhg.2012.09.001. 23084291 PMC3487136

[10] A. Alodaib , N. Sobreira , W. A. Gold , et al., “Whole‐Exome Sequencing Identifies Novel Variants in PNPT1 Causing Oxidative Phosphorylation Defects and Severe Multisystem Disease,” European Journal of Human Genetics 25, no. 1 (2017): 79–84, 10.1038/ejhg.2016.128. 27759031 PMC5159763

[11] A. Hosseini Bereshneh , Z. Rezaei , E. Jafarinia , et al., “Crystallographic Modeling of the PNPT1:C.1453A>G Variant as a Cause of Mitochondrial Dysfunction and Autosomal Recessive Deafness; Expanding the Neuroimaging and Clinical Features,” Mitochondrion 59 (2021): 1–7, 10.1016/j.mito.2021.03.012. 33812062

[12] A. Pennisi , A. Rötig , C.‐J. Roux , et al., “Heterogeneity of PNPT1 Neuroimaging: Mitochondriopathy, Interferonopathy or Both?,” Journal of Medical Genetics 59, no. 2 (2022): 204–208, 10.1136/jmedgenet-2020-107367. 33199448

[13] B. Lin and R. Goldbach‐Mansky , “Pathogenic Insights From Genetic Causes of Autoinflammatory Inflammasomopathies and Interferonopathies,” Journal of Allergy and Clinical Immunology 149, no. 3 (2022): 819–832, 10.1016/j.jaci.2021.10.027. 34893352 PMC8901451

[14] I. Melki and M.‐L. Frémond , “Type I Interferonopathies: From a Novel Concept to Targeted Therapeutics,” Current Rheumatology Reports 22, no. 7 (2020): 32, 10.1007/s11926-020-00909-4. 32548765

[15] L. O. Mendonça and M.‐L. Frémond , “Interferonopathies: From Concept to Clinical Practice,” Best Practice & Research. Clinical Rheumatology 38, no. 3 (2024): 101975, 10.1016/j.berh.2024.101975. 39122631

[16] J. Hadjadj , M.‐L. Frémond , and B. Neven , “Emerging Place of JAK Inhibitors in the Treatment of Inborn Errors of Immunity,” Frontiers in Immunology 12 (2021): 717388, 10.3389/fimmu.2021.717388. 34603291 PMC8484879

[17] H. M. Hoffman and L. Broderick , “JAK Inhibitors in Autoinflammation,” Journal of Clinical Investigation 128, no. 7 (2018): 2760–2762, 10.1172/JCI121526. 29889100 PMC6025991

[18] W. Li , W. Wang , W. Wang , et al., “Janus Kinase Inhibitors in the Treatment of Type I Interferonopathies: A Case Series From a Single Center in China,” Frontiers in Immunology 13 (2022): 1–11, 10.3389/fimmu.2022.825367. PMC8995420 35418997

[19] Y. Liu , A. A. Jesus , B. Marrero , et al., “Activated STING in a Vascular and Pulmonary Syndrome,” New England Journal of Medicine 371, no. 6 (2014): 507–518, 10.1056/NEJMoa1312625. 25029335 PMC4174543

[20] G. A. M. Sanchez , A. Reinhardt , S. Ramsey , et al., “JAK1/2 Inhibition With Baricitinib in the Treatment of Autoinflammatory Interferonopathies,” Journal of Clinical Investigation 128, no. 7 (2018): 3041–3052, 10.1172/JCI98814. 29649002 PMC6026004

[21] S. Richards , N. Aziz , S. Bale , et al., “Standards and Guidelines for the Interpretation of Sequence Variants: A Joint Consensus Recommendation of the American College of Medical Genetics and Genomics and the Association for Molecular Pathology,” Genetics in Medicine 17, no. 5 (2015): 405–424, 10.1038/gim.2015.30. 25741868 PMC4544753

[22] M. T. Lam , A. Basu , K. E. Brodeur , et al., “Clinically Validated Assay for Rapid Determination of Type I and Type II Interferon Activity in Systemic Inflammatory Diseases,” Journal of Allergy and Clinical Immunology (2026), 10.1016/j.jaci.2026.01.021. 41638262

[23] V. Matteo , H. Zeric , E. Loricchio , et al., “SIGLEC‐1 Expression on Monocytes as a Diagnostic Biomarker in Pediatric Type I Interferon‐Mediated Diseases,” Journal of Allergy and Clinical Immunology (2026), 10.1016/j.jaci.2026.02.007. 41720274

[24] N. Sakumura , T. Yokoyama , M. Usami , et al., “CD169 Expression on Monocytes as a Marker for Assessing Type I Interferon Status in Pediatric Inflammatory Diseases,” Clinical Immunology 250 (2023): 109329, 10.1016/j.clim.2023.109329. 37061149

[25] K. Cetin Gedik , L. Lamot , M. Romano , et al., “The 2021 European Alliance of Associations for Rheumatology/American College of Rheumatology Points to Consider for Diagnosis and Management of Autoinflammatory Type I Interferonopathies: CANDLE/PRAAS, SAVI, and AGS,” Arthritis and Rheumatology 74, no. 5 (2022): 735–751. 35315249 10.1002/art.42087

[26] M.‐L. Frémond , M. Hully , B. Fournier , et al., “JAK Inhibition in Aicardi‐Goutières Syndrome: A Monocentric Multidisciplinary Real‐World Approach Study,” Journal of Clinical Immunology 43, no. 6 (2023): 1436–1447, 10.1007/s10875-023-01500-z. 37171742 PMC10175907

[27] N. K. Bagri , C. Chew , and A. V. Ramanan , “Scope of JAK Inhibitors in Children: Recent Evidence and Way Forward,” Pediatric Drugs 25, no. 6 (2023): 635–647, 10.1007/s40272-023-00594-7. 37775678

[28] H. I. Brunner , J. D. Akikusa , E. Al‐Abadi , et al., “Safety and Efficacy of Tofacitinib for the Treatment of Patients With Juvenile Idiopathic Arthritis: Preliminary Results of an Open‐Label, Long‐Term Extension Study,” Annals of the Rheumatic Diseases 83, no. 11 (2024): 1561–1571, 10.1136/ard-2023-225094. 38849152 PMC11503147

[29] Y. Du , M. Liu , P. A. Nigrovic , F. Dedeoglu , and P. Y. Lee , “Biologics and JAK Inhibitors for the Treatment of Monogenic Systemic Autoinflammatory Diseases in Children,” Journal of Allergy and Clinical Immunology 151, no. 3 (2023): 607–618, 10.1016/j.jaci.2022.12.816. 36707349 PMC9992337

[30] N. Ruperto , H. I. Brunner , O. Synoverska , et al., “Tofacitinib in Juvenile Idiopathic Arthritis: A Double‐Blind, Placebo‐Controlled, Withdrawal Phase 3 Randomised Trial,” Lancet 398, no. 10315 (2021): 1984–1996, 10.1016/S0140-6736(21)01255-1. 34767764

[31] S. Talasila , E. Lee , E. M. Teichner , E. C. Siegfried , and S. R. Jackson Cullison , “Analysis of Publicly Available Adverse Events Reported for Pediatric Patients Treated With Janus Kinase Inhibitors,” Pediatric Dermatology 41, no. 6 (2024): 1040–1046, 10.1111/pde.15721. 39235110

[32] S. Kataoka , N. Kawashima , Y. Okuno , et al., “Successful Treatment of a Novel Type I Interferonopathy due to a de Novo PSMB9 Gene Mutation With a Janus Kinase Inhibitor,” Journal of Allergy and Clinical Immunology 148, no. 2 (2021): 639–644, 10.1016/j.jaci.2021.03.010. 33727065

[33] D. Shen , X. Fan , Q. Zhou , X. Xu , and M. Lu , “Use of Tofacitinib for Infant‐Onset STING‐Associated Vasculopathy: A Case Report From China,” Medicine 101, no. 48 (2022): e31832, 10.1097/MD.0000000000031832. 36482559 PMC9726360

[34] Z. Boyadzhieva , N. Ruffer , G. Burmester , A. Pankow , and M. Krusche , “Effectiveness and Safety of JAK Inhibitors in Autoinflammatory Diseases: A Systematic Review,” Frontiers in Medicine 9 (2022): 930071, 10.3389/fmed.2022.930071. 35833101 PMC9271622

[35] A. Pin , A. Tesser , S. Pastore , et al., “Biological and Clinical Changes in a Pediatric Series Treated With Off‐Label JAK Inhibitors,” International Journal of Molecular Sciences 21, no. 20 (2020): 7767, 10.3390/ijms21207767. 33092242 PMC7590237

[36] E. Morava , I. Elsharkawi , and T. Kozicz , “Reframing Primary Mitochondrial Disease as a Sterile Interferonopathy,” Molecular Genetics and Metabolism 146, no. 1–2 (2025): 109217, 10.1016/j.ymgme.2025.109217. 40753693

[37] A. Lepelley , E. della Mina , E. van Nieuwenhove , et al., “Enhanced cGAS‐STING–Dependent Interferon Signaling Associated With Mutations in ATAD3A,” Journal of Experimental Medicine 218, no. 10 (2021): e20201560, 10.1084/jem.20201560. 34387651 PMC8374862

[38] D. G. Calame , E. K. Wiener , F. Gavazzi , et al., “PTPN1—Related Autoinflammation Is a Common Cause of Aicardi‐Goutières Syndrome With Reduced Penetrance,” 2026, 10.64898/2026.03.27.26345228.

[39] S. Braidotti , R. M. Ferraro , R. Franca , et al., “Pharmacological Evaluation of Drug Therapies in Aicardi‐Goutières Syndrome: Insights From Patient‐Derived Neural Stem Cells,” Frontiers in Pharmacology 16 (2025): 1549183, 10.3389/fphar.2025.1549183. 40183101 PMC11966042

[40] G. Marinella , Y. Vaia , D. Politano , et al., “Efficacy of JAK1/2 Inhibitors in AGS Genes‐Related Interferonopathies: A Multicenter Retrospective Observational Study With Treated vs Untreated Comparison,” Molecular Genetics and Metabolism 148, no. 2 (2026): 109907, 10.1016/j.ymgme.2026.109907. 41871482

[41] V. Boczonadi , B. Bansagi , and R. Horvath , “Reversible Infantile Mitochondrial Diseases,” Journal of Inherited Metabolic Disease 38, no. 3 (2015): 427–435, 10.1007/s10545-014-9784-6. 25407320

[42] M. A. Walker , M. Miranda , A. Allred , and V. K. Mootha , “On the Dynamic and Even Reversible Nature of Leigh Syndrome: Lessons From Human Imaging and Mouse Models,” Current Opinion in Neurobiology 72 (2022): 80–90, 10.1016/j.conb.2021.09.006. 34656053 PMC8901530

[43] G. Zhu , B. Didry‐Barca , L. Seabra , et al., “Autoinflammatory Encephalopathy due to PTPN1 Haploinsufficiency: A Case Series,” Lancet Neurology 24, no. 3 (2025): 218–229, 10.1016/S1474-4422(24)00526-X. 39986310 PMC7617446

